# Metal Exsolution to Enhance the Catalytic Activity of Electrodes in Solid Oxide Fuel Cells

**DOI:** 10.3390/nano10122445

**Published:** 2020-12-07

**Authors:** Tianyu Cao, Ohhun Kwon, Raymond J. Gorte, John M. Vohs

**Affiliations:** Department of Chemical and Biomolecular Engineering, University of Pennsylvania, 34th Street, Philadelphia, PA 19104, USA; caot@seas.upenn.edu (T.C.); ohhun@seas.upenn.edu (O.K.); vohs@seas.upenn.edu (J.M.V.)

**Keywords:** solid oxide fuel cell, catalysis, ceramic anode, metal exsolution

## Abstract

Exsolution is a novel technology for attaching metal catalyst particles onto ceramic anodes in the solid oxide fuel cells (SOFCs). The exsolved metal particles in the anode exhibit unique properties for reaction and have demonstrated remarkable stabilities under conditions that normally lead to coking. Despite extensive investigations, the underlying principles behind exsolution are still under investigation. In this review, the present status of exsolution materials for SOFC applications is reported, including a description of the fundamental concepts behind metal incorporation in oxide lattices, a listing of proposed mechanisms and thermodynamics of the exsolution process and a discussion on the catalytic properties of the resulting materials. Prospects and opportunities to use materials produced by exsolution for SOFC are discussed.

## 1. Introduction

The most commonly used material for the fuel electrodes (the anode in Solid Oxide Fuel Cells (SOFCs) and the cathode in Solid Oxide Electrolysis Cells (SOECs)) in Solid Oxide Cells (SOCs) is a physical mixture of metallic Ni and the electrolyte material, typically either Yttria-Stabilized Zirconia (YSZ) [1] or Gd-doped Ceria (GDC) [2]. These Ni-based ceramic–metallic (cermet) composites are used because they exhibit excellent performances in syngas, a mixture of CO and H_2_. For electrode-supported cells, the Ni cermet also provides mechanical strength. However, Ni cermets have limitations. For example, they tend to fracture upon reoxidation due to expansion of the Ni upon forming NiO [3]. Additionally, in the presence of hydrocarbons or during operation in fuels with low H_2_O:C ratios, Ni catalyzes the formation of carbon fibers, which can plug the pores and again cause cell fracture [4]. Conventional Ni-based cells are also sensitive to sulfur impurities in the fuel [5].

To address some of the drawbacks of Ni cermet anodes, electronically conductive oxides, which do not catalyze formation of carbon fibers and are resistant to forming sulfides, have been explored as an alternative to Ni as the electronically conductive component. Volume changes upon oxidation or reduction are not significant for most oxides, making ceramic electrodes stable against expansion over a wide range of P(O_2_). While the mechanical properties of ceramic electrodes are less favorable than those of Ni cermets, strength issues can be addressed using electrolyte-supported cells or by adding other support structures [6,7]. Therefore, some of the most important problems associated with Ni cermets can be solved by using fuel electrodes based on electronically conducting oxides. Unfortunately, the catalytic activities of oxides for fuel oxidation are not comparable to that of Ni or other transition metals from groups 9 or 10 in the periodic table. In order to achieve high performance with ceramic electrodes, it is therefore necessary to add small amounts of a catalytic metal [8]. While decorating metals onto the ceramic electrodes for catalytic purposes is simple to do by solution-phase impregnation, sintering of the metal catalyst and the resulting loss in catalytic activity are severe problems at SOC operating temperatures [9].

**Figure 1:**
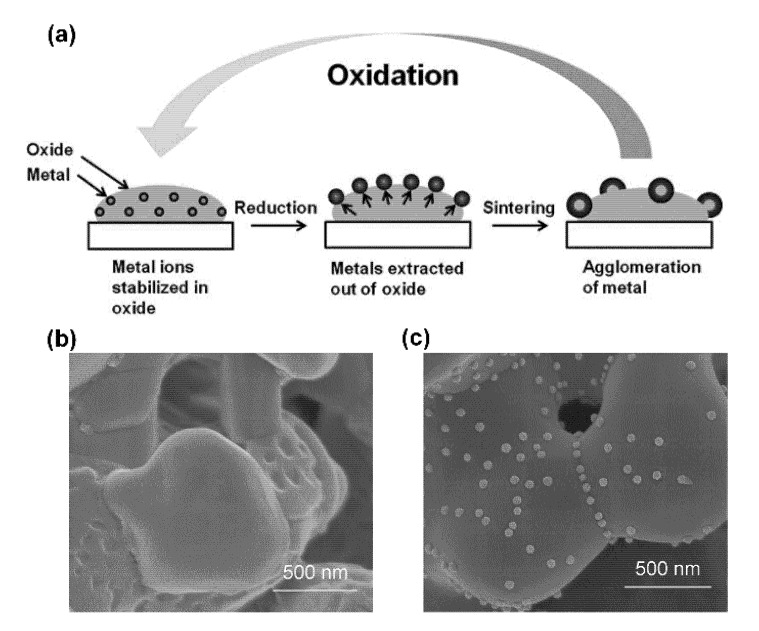
(**a**) Schematic of exsolution process; figure reprinted from Ref. [12] with permission from The Royal Society of Chemistry, 2012; Scanning electron microscope (SEM) photos of exsolution materials before (**b**) and after (**c**) metal exsolution; figures reprinted from Ref. [13] with permission from Elsevier, 2016.

A potential solution to the sintering problem is to incorporate what has been referred to as “intelligent” catalysts into the conductive ceramics. In this concept, which is borrowed from the automotive emissions-control literature [10], the supported metal catalyst is formed via exsolution from an oxide host (usually a perovskite-type of oxide). There is evidence that supported metal catalysts formed in this way exhibit high stability, which could help address the sintering issue, and may also be less prone to forming carbon filaments when operating in fuels with lower H_2_O:C ratios [11]. The concept of exsolution is shown schematically in Figure 1a and Scanning Electron Microscope (SEM) images of supported metal particles formed using this method are shown in Figure 1b,c.

Madsen et al. [14] appear to have been the first to apply this concept to an SOFC and to demonstrate that metal exsolution can lead to enhanced electrode performance. These researchers incorporated Ru into the lattice of Sr-doped LaCrO_3_ (La_0.8_Sr_0.2_Cr_0.82_Ru_0.18_O_3−δ_) and used this material as an SOFC anode. Ru cations in the oxide were reduced to Ru^0^ upon exposure to humidified H_2_ at 1073 K, forming nanoparticles with diameters of approximately 5 nm. The presence of exsolved Ru significantly lowered the impedance of the anode. Since this early work, many other examples of electrode promotion by exsolution have been reported. In this paper, we provide a brief review of this literature.

## 2. Towards an Optimal Anode for SOFC

**Figure 2:**
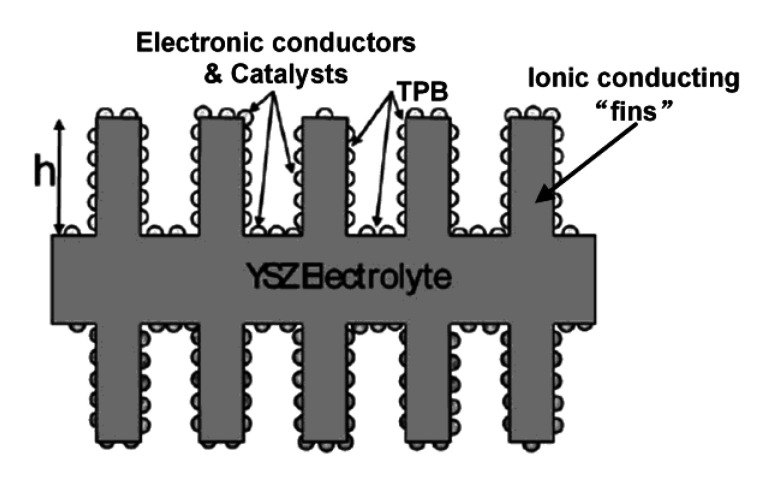
Schematic demonstrating an idealized structure of “fin-like” electrodes in a solid oxide fuel cells (SOFC); figure reprinted from Ref. [16] with permission from John Wiley and Sons, 2009.

Before describing in detail exsolution and its application to SOFC anodes, it is useful to first review the requirements for high-performance ceramic electrodes and to consider the structure of an optimized conventional Ni cermet. Electrodes in a SOC must have both high electronic and oxygen-ion conductivities. This need for Mixed Ionic and Electronic Conductivity (MIEC) is usually achieved by using a physical mixture of the two types of conductors, e.g., Ni and YSZ, in the case of Ni-YSZ cermets. The factors that lead to good performance for Ni-YSZ composites have been analyzed theoretically by Tanner et al. [15], and a diagram of the ideal structure is shown in Figure 2.

To maximize the concentration of triple-phase-boundary (TPB) sites where the electrochemical reactions occur, the electrodes needs to have “fins” or other porous structures of the ion-conducting material that extend away from the electrolyte. The presence of these fins in the functional layer of the anode allows O^2−^ ions to diffuse more deeply into the electrode, increasing the surface area available for reaction and decreasing the resistance due to surface reactions. In a Ni-YSZ cermet, the YSZ fins are ideally coated with Ni, which provides the electronic conductivity and catalytic sites. Because this structure is more optimally achieved when the Ni cermet is co-fired with the electrolyte, anode-supported SOFCs typically exhibit much lower anode overpotentials than what is observed in electrolyte-supported cells. 

Electronic conductivity can be achieved in composites with electronic conductors other than Ni. For example, Cu has been investigated as an electronic conducting material in the anode of SOFCs that oxidize hydrocarbons directly, without first reforming to form a hydrogen rich gas [17,18,19]. This is possible because Cu, unlike Ni, does not catalyze the formation of carbon fibers. A major problem with Cu electrodes, however, is that Cu sinters at relatively low temperatures. The implication for the ideal structure in Figure 2 is that the pathway for electron transport is unstable, since the Cu forms discrete disconnected particles that no longer percolate the structure [20]. Although Ni resists sintering much more effectively, similar coarsening issues are a problem for Ni cermets as well [21].

As noted above, another promising approach is to replace the metal current collector with an electronically conductive ceramic material. Because the surface energies of metal oxides are much lower than those of metals, oxides are inherently more resistant to sintering. Therefore, electrodes based on conductive ceramics are expected to be significantly more stable. Corre et al. have even reported that La_0.75_Sr_0.25_Cr_0.5_Mn_0.5_O_3−δ_ (LSCM) spontaneously “spreads” into a porous film on YSZ following reduction at operating conditions [22]. Thus, at least in some cases, electronically conductive ceramic coatings tend to form a structure that is very close to that of the ideal situation for maximizing TPB sites.

As mentioned earlier, the catalytic activities of oxides for oxidation of H_2_ and CO are low, so that good performance can only be achieved when an additional catalyst is added [8]. Although metallic catalyst particles can be added by infiltration of metal salts in solution, the metal particles formed in this way tend to be unstable. For example, when Pd particles were added to LSCM–YSZ composites, the performance was initially excellent; but treatment at 1173 K, a temperature that is still modest by SOFC standards, significantly degraded the performance due to sintering of the metal particles and the resulting loss of active surface area [23]. Therefore, to make ceramic anodes viable, it is essential that the metal particles be stabilized.

Maintaining metal dispersion, defined as the fraction of metal atoms at the surface of the nanoparticles, has always been a challenge in the use of supported metal catalysts. This is especially true for automotive emissions-control catalysts because of the harsh conditions to which these materials are exposed [24,25]. Automotive catalysts can experience temperatures in excess of 1000 °C, in high-steam environments that cycle between reducing and oxidizing conditions [26]. In an attempt to address sintering issues, Tanaka and his co-workers at Daihatsu Motor Company developed the concept of “intelligent” catalysts [10,27,28,29,30]. In their studies of Pd-doped perovskites, it was observed that, upon high-temperature reduction, Pd could be reduced to its metallic form and “ex-solved” to the surface of the perovskite (see Figure 1). The behavior was found to be reversible, with Pd being restored to the perovskite lattice following high-temperature oxidation. Moreover, the authors reported that Pd remained highly dispersed after accelerated aging. Results for Rh- and Pt-based perovskites shows they exhibited similar behaviors [10]. 

Strongly reducing conditions (e.g., 1073 K in relatively dry H_2_ [11]) are typically required to bring metals to the surface of these exsolution catalysts. This requirement represents a potential limitation for many catalytic applications, where treating the catalyst this harshly can affect other properties, such as loss of support surface area. However, it is worth noting that it is much easier to change the effective P(O_2_) in SOCs by simply applying a reducing overpotential on the electrode [31]. This very interesting concept of “electrochemical switching” has been demonstrated by several authors in different electrochemical systems [32,33,34,35]. 

A great deal of additional research has been performed since this early work on emissions-control catalysis, including work on using these exsolution systems in solid oxide fuel cells [36,37,38] and hydrocarbon-reforming catalysis [11,39,40]. Many of those investigations focused on the exsolution process itself and are therefore relevant to all applications [41,42,43]. Here, we will restrict our consideration to the use of the exsolution concept to SOFC electrodes and describe some of the important features associated with metal exsolution in this context. We only consider cases where the cations of the catalytic metal can be definitely shown to be part of the crystalline lattice of the host oxide in the oxidized form of the material.

## 3. Conductive Oxides and Exsolution Hosts

**Figure 3:**
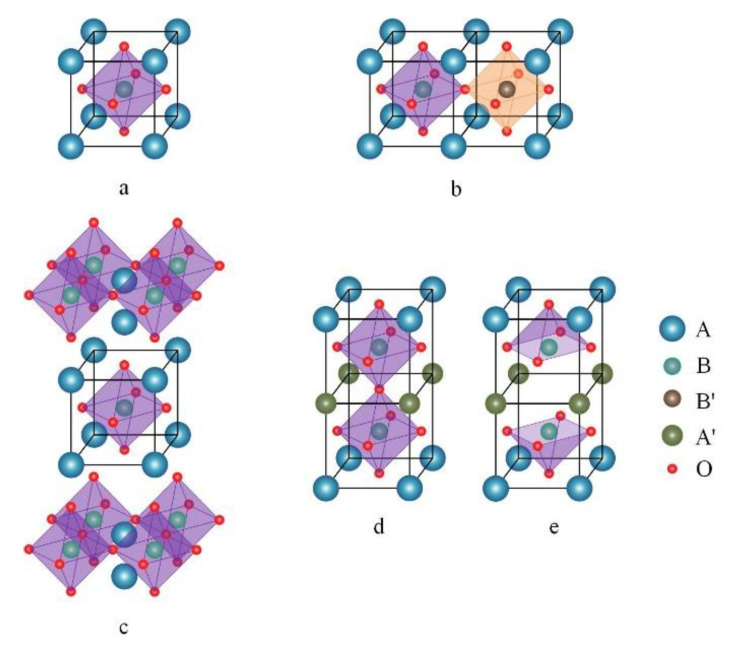
Unit cells of perovskites in this review: (**a**) ABO_3_ structure of simple perovskite; (**b**) A_2_BB’O_6_ of double perovskite, volume of its unit cell is twice as that of the simple perovskite; (**c**) Ruddlesden–Popper (RP) perovskite, with one layer of ABO_3_ cells sandwiched in the middle; AA’B_2_O_5+δ_ layered double perovskite, (**d**) oxidized; (**e**) reduced (δ~0). Spheres of different colors denotes different sites. Black frame in the ABO_3_ unit cell is a guide for the eyes.

Many conductive ceramics have perovskite or perovskite-related structures as shown in Figure 3 and are therefore are ideally suited for use in SOC electrodes that take advantage of the exsolution concept. The unit cell of a simple perovskite, ABO_3_, is presented in Figure 3a. The B-site cations reside at the center of the unit cell, coordinated by six oxygen atoms forming a BO_6_ octahedron, while the A-site cations sit at the corners of the unit cell. The sum of the charges on the A and B cations must be six; although the perovskite structure allows some mismatch in the lengths of the A-O and B-O bonds, this mismatch is limited in that the Goldschmidt tolerance factor, *t,* must be between 1 and 0.75. This tolerance factor is a function of the radii of the constituent ions, as shown in Equation (1).

(1) $$t=\frac{r_{O}+r_{A}}{\sqrt{2}\left(r_{O}+r_{B}\right)}$$

Here, *r*_A_, *r*_B_ and *r*_O_ are the ionic radii of the A-site cation, the B-site cation and the oxygen anions, respectively. Various metal cations can be substituted into the lattice so as long as the guest cation meets the requirement of crystal stability. Substitution of cations with different or variable charges can be used to introduce electronic or ionic conductivity, as well as produce oxygen vacancies in the lattice.

Electronic conductivity in perovskites results when the B site has multivalent cations, such as Cr^3+/4+^, Ti^3+/4+^, Fe^2+/3+^, Mn^2+/3+/4+^, etc. [44,45] The ability of these atoms to change valance allows movement of charge through the lattice. Doping the A sites with divalent cations greatly enhances the ionic conductivity by introducing oxygen vacancies and stabilizing reduction of B-site cations. A complicating factor with conductive oxides is that their conductivities are usually a strong function of P(O_2_), which, under reducing conditions, is determined by the H_2_:H_2_O ratio and temperature. For SOFC anodes, n-type conductivity is preferred, because it tends to increase in more reducing atmospheres [46]. 

In addition to simple perovskites, more complex structures that contain BO_6_ octahedra also find application in SOFCs. Double perovskites, shown diagrammatically in Figure 3b, are a simple variant in which the B sites contain two different metal cations, B and B’, in an alternating arrangement. Double perovskites, such as doped LaCr_0.5_Mn_0.5_O_3_ [47], can take on properties intermediate between the two end points, such as providing the stability of LaCrO_3_ in reducing conditions and the higher conductivity of LaMnO_3_. Another common variant, the Ruddlesden–Popper perovskite (RP-perovskite), is often used for electrode applications because materials with this structure often have relatively high ionic conductivities. The RP-perovskite structure, shown in Figure 3c, is made up of slabs of BO_6_ octahedra stacked together, with A-site cations sandwiched between two neighboring slabs. Ionic conductivity is increased with this structure because the activation energy for oxygen anions to move along these [AO] layers is decreased [48]. 

Direct synthesis of the RP-perovskite phase usually requires high temperatures [49,50]. Alternatively, this phase can sometimes be achieved by reductive treatments when the starting material, either a simple [51] or double perovskite [52], contains reducible cations at the B sites that can be removed from the lattice. The RP-perovskite phase is then induced when the reducible cations leave the lattice. Producing the RP-perovskites through this phase transition results in a material with excess A-site cations.

A fourth related structure is the layered double perovskite—Figure 3d,e. These materials always contain barium cations, which are required in order to facilitate the formation of the layers, due to their large size. The standard structure of layered double perovskites (AA’B_2_O_5+δ_) consists of five layers of ordered oxides stacked in sequence: [AO]-[BO_2_]-[A’O_δ_]-[BO_2_]-[AO], where δ is between 0 and 1. The value of δ can be fairly large under the oxidizing atmospheres; for example, the value of δ in PrBa_0.8_Ca_0.2_Mn_2_O_5+δ_ (PBCM) decreases from ~0.8 to ~0 when the oxygen partial pressure drops from 10^−2^ to 10^−25^ atm [53]. Under reducing conditions, the unit cell of PBCM will transition from that shown in Figure 3d to that presented in Figure 3e. Removal of this large amount of oxygen from the lattice creates defective [A’O_δ_] layers, which can serve as diffusive channels for ion transport. The double-perovskite structure has been reported to exhibit high ionic conductivities [54,55]. The energy barrier for exsolution of metal cations can also be lower in this structure compared to that of simple perovskites [48,56].

Perovskite-type of materials are not the only conductive oxides which are able to host metal cations for exsolution. For example, Adijanto et al. [57] used rare-earth vanadates, which have very high electronic conductivities under reducing conditions, as the host. Because V ions undergo deep reduction, from +5 to +3, the doped vanadates transform from a zircon-type crystal structure to a perovskite-type structure after exposure to a reducing atmosphere [58]. Metal particles were shown to exsolve from the oxide during this phase transition [12]. Mixed oxides with rutile structures have also been shown to exhibit exsolution characteristics [59].

## 4. Manner of Lattice Substitution

As mentioned earlier, we consider only cases where the metal cations can enter the lattice of the host oxide and exclude situations in which the oxidized metal catalyst and the host exist as a simple physical mixture. Demonstrating that the dopant catalytic metal cations enter the host lattice is often nontrivial and typically involves spectroscopic evidence for lattice substitution [10] or a shift in the host lattice parameter [57]. Substitution of cations into the lattice of a host oxide is also specific to the composition of both the metal cations and the host oxide, and not all metal cations can be incorporated into all ceramics, including those with perovskite structures. This was demonstrated by Tanaka et al. [10], who reported this specificity in a study of Pd, Pt and Rh in a series of simple perovskites using X-Ray Absorption Spectroscopy (XAS). Among their findings, they observed that Pd could enter the LaFeO_3_ lattice but not that of CaTiO_3_, while Pt and Rh could become part of both perovskite lattices. 

Often, there is uncertainty about whether or not the metal cations undergo lattice substitution in the host oxide. For example, diffraction studies of Pd and LaCrO_3_ revealed numerous secondary peaks in the doped perovskites, including ones associated with phases identified as PdO, LaPdO_3_ and La_4_PdO_7_ [60]. Based on Scanning Transmission Electron Microscopy (STEM) results, said studies suggested most of the Pd added to the LaCrO_3_ went into secondary phases, rather than being incorporated as a dopant into the LaCrO_3_ phase [60]. As a result, the nanosized Pd particles formed by reduction of the PdO-LaCrO_3_ system should probably be considered separately from other exsolution systems, since the mechanism by which metal nanoparticles are formed upon high-temperature reduction could be very different.

There is also evidence that metal cations can substitute into either the A site or B site of the lattice and that exsolution can take place in both A site-deficient and stoichiometric perovskites [61]. For example, the work of Tanaka et al. [10] indicated that Pt and Rh cations substitute primarily for Ti^4+^ at the B sites when they are incorporated into CaTiO_3_. Similarly, most reducible cations, including Ni^2+^, Co^2+/3+^ etc., are usually considered to occupy the B sites of simple perovskites such as LaFeO_3_. However, A-site substitution is possible when the radius of the A-site cation is similar in size to that of the dopant cations and some metals appear to occupy both types of sites. For example, using STEM measurements of the Pt-CaTiO_3_ system with small-angle tilting, Zhang et al. [62] showed that 30 to 40% of the Pt are in the A sites, with the rest substituting for B sites. These authors also reported that, upon reduction, Pt from the sub-nanometer Pt clusters that remained embedded in the oxide would primarily occupy the A sites upon reoxidation.

## 5. Mechanisms and Energetics of the Exsolution Process

The details of the exsolution process may vary with the specific system. In at least some cases, metal clusters are formed in the bulk of the host oxide upon reduction, after which the particles are forced to the surface by strain in the host oxide [42,62,63,64]. In other cases, metal cations may diffuse to the surface, be reduced and form particles by their interaction with other metal cations. We will discuss each of these mechanisms separately. 

The fact that metal particles can form in the bulk of the host perovskite oxide upon reduction has been clearly demonstrated in some cases. For example, STEM measurements performed for Pt-doped CaTiO_3_ films clearly show that some of the Pt particles remained beneath the surface [62]. In a TEM study of Ni particles formed from a slab of La_0.2_Sr_0.7_Ti_0.9_Ni_0.1_O_3−δ_, cross-sectional images revealed that most of the Ni particles remained embedded in the bulk [65]. In still another study of La_0.2_Sr_0.7_Ti_0.9_Ni_0.1_O_3−δ_ surfaces, pits were formed in the surface during the initial stages of reduction, with metal particles emerging from those pits later in the reduction process [42]. The particles remained partially embedded within the surface and this appears to stabilize them against sintering to some extent. 

Based on the observation that metal particles emerged from pits during the reduction process, Oh et al. [42] concluded that metal particles are first formed in the bulk of the host oxide and then exsolve as fully formed particles. Exsolution is hypothesized to be driven by the fact that the system is at higher energy when the particles are in the bulk due to the strain field within the host, so that exsolution lowers the total energy of the system. An interesting conclusion from the strain-field calculation was that only particles that are close to the free surface, within approximately three times the particle radius, can achieve relaxation by emerging from the host. 

Kim et al. [65] also analyzed the energetics of Ni particles forming in the bulk of the La_0.2_Sr_0.7_Ti_0.9_Ni_0.1_O_3−δ_ host. They considered Ni particles that were only partially exposed to the gas phase upon reduction, with the remaining particles partially in the oxide host. Their model revealed that innate mechanical properties, such as the Young’s modulus and Poisson’s ratio of both the metal and the oxide, play important roles in the process of exsolution. By changing the strain field, differences in the mechanical properties lead to differences in the number, radius and depth of the anchored particles.

The second possible mechanism for exsolution involves reduction of metal cations that diffuse to the surface of the host. For example, in their STEM study of Ni particles forming on La_0.43_Ca_0.37_Ti_0.94_Ni_0.06_O_3_, Neagu et al. [66] hypothesized that the Ni^2+^ cations must migrate to the surface of the host oxide before being reduced by H_2_. Their evidence for this was that Ni particles only formed on the surface of the sample and remained fixed at the locations where they initially nucleated. Gao et al. [13] evaluated the surface reduction mechanism for the closely related La_0.4_Sr_0.4_Sc_0.9_Ni_0.1_O_3−δ_ exsolution system. They concluded that the reduction of surface Ni^2+^ cations nucleated further growth through migration and reduction of additional Ni^2+^ from the bulk and that the growth rate for the exsolved Ni^0^ particles could be controlled by the strain between the particle and its host, the reduction kinetics of Ni^2+^ and mass transport of Ni^2+^ from the bulk.

The surface reduction mechanism has also been proposed for the layered double perovskites. For example, Kwon et al. [36] proposed a co-exsolution mechanism to describe the egress of metals from PrBaMn_1.7_M_0.3_O_5+δ_ (M=Ni, Co). In this mechanism, metal cations in the bulk of the material exsolve to the surface together with a nearby oxygen vacancy. The metal cations on the surface are further stabilized by the creation of adjacent oxygen vacancies as part of the reduction process. This mechanism may be preferred in layered double perovskites due to the large concentration of lattice oxygen vacancies in these materials.

It is important to recognize that the host oxide will undergo a lattice reorganization process when metal cations undergo exsolution, no matter what mechanism is operative. Removal of metal cations from the lattice creates vacancies that will lead to distortions in the crystal structure or possibly even a general loss of crystallinity in the oxide [67,68]. For instance, exsolution of B-site cations leaves excess A-site cations in the oxide. As discussed earlier, this can lead to transformation of a perovskite phase to an RP-phase [41]. In other cases, structural changes may be even more severe [69]. 

One method to solve this problem, referred to as topotactic ion exchange, involves providing an external source of metal cations to replace the vacancies. Joo et al. [70] reported an example of this with the layered double-perovskite phase (PBMCo, PrBaMn_1.7_Co_0.3_O_5+δ_). After adding Fe cations onto the surface of fresh PBMCo by infiltration, Co cations were found to exsolve from the lattice under reducing atmospheres while the deposited Fe cations filled the vacancies left over by the exsolution. The addition of Fe increased the number density of exsolved nanoparticles from 24 particles per μm^2^ to 103 μm^−2^. The exsolved Co also formed a Co-Fe alloy with the remaining Fe. The increased driving force for this ion-exchange process is the difference in the exsolution energies between the two cations, with the external cation being the one that is more difficult to reduce. A paper from the same group also used Fe as the external cation to make Ni-Fe alloy particles [71]. In addition to these cases on the layered double-perovskite phase, topotactic exsolution is also viable on simple perovskite systems [72].

**Figure 4:**
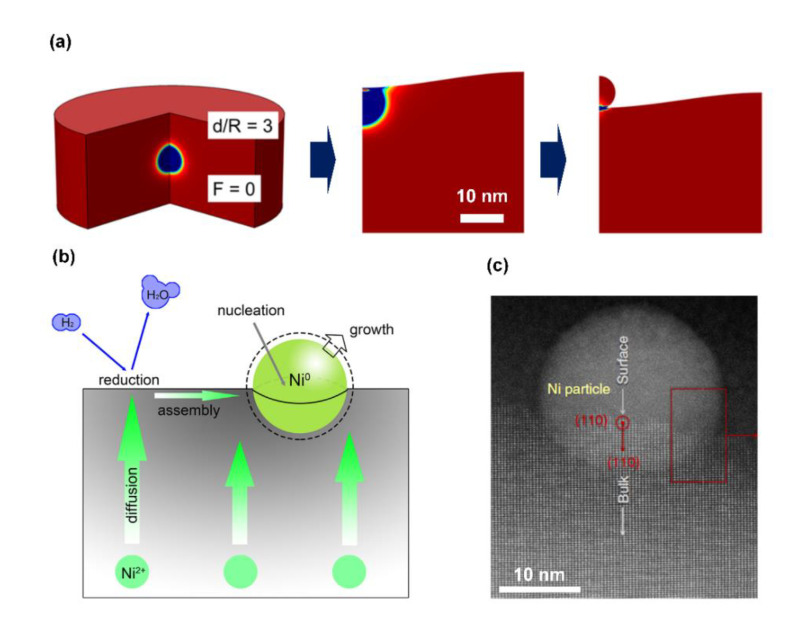
(**a**) Schematic of a spherical Ni particle exsolves from the bulk of its host and forms an embedded structure, figure reprinted with permission from Ref. [42]. Copyright (2015) American Chemical Society; (**b**) schematic of the “surface reduction” mechanism, figure reprinted from Ref. [13] with permission from Elsevier; (**c**) a Scanning Transmission Electron Microscopy (STEM) photo of an exsolved Ni particle embedded in its La_0.52_Sr_0.28_Ni_0.06_Ti_0.94_O_3−δ_ host, figure reprinted from Ref. [11].

A distinguishing characteristic of many exsolution systems is that the exsolved metal particles form a unique interface with the surface of the oxide host in which the particle appears to be partially embedded or socketed. This is shown schematically in Figure 4a for the case where metal particles originally form in the bulk before being ejected to the surface. The socketed structure results from the balancing of the lattice strain due to the metal particle and the surface free energy. In cases where the surface reduction mechanism is operative, particle embedding could still occur as a consequence of particle growth. According to Neagu et al. [66], Ni particles on the surface grow epitaxially and push the adjacent oxide laterally apart to make room for growth. Hence, stress in the surrounding oxide lifts the surface to form a volcano-shaped socket. Schematics of these two exsolution mechanisms and a STEM photo of the socketed exsolved metal particle are presented in Figure 4c.

### Thermodynamic Considerations

The thermodynamics of oxidation and reduction of pure metals is well understood. For example, the thermodynamics of Ni oxidation can be described by the reaction, Ni+ 12O2=NiO, for which the equilibrium constant, $K_{NiO}$, is equal to PO2−12. $K_{NiO}$ is determined from ∆G of the oxidation reaction. However, if the Ni^2+^ ions exist in a perovskite lattice, ΔG of the oxidation reaction is expected to be different from that of bulk NiO [31]. Mao et al. [73] provided a demonstration of this in the Ni-LaFeO_3_ system by measuring oxidation equilibrium constants for Ni particles on the surface of LaFeO_3_ thin films. The equilibrium constant was found to decrease by five orders of magnitude compared to that of bulk NiO. The difference was attributed mainly to the entropic component of ΔG.

Given the limitations of using bulk properties to predict exsolution and difficulties in directly measuring the thermodynamics of this process, computational chemistry methods, such as Density Functional Theory (DFT), are more frequently used to provide insights into the energetics of exsolution. DFT calculations typically use lattice models that contain only tens of atoms arranged into a few unit cells of the crystal structure. Even with this limitation, they provide useful insights into the interactions between the various ions and into the energetics of the exsolution process. Due to their limited scale however, DFT models cannot study the metal nucleation process itself.

As an example of this kind of study, Hamada et al. [74] used DFT to investigate the exsolution of Rh, Pt and Pd that had been doped into LaFeO_3_. Their model consisted of five two-dimensional, FeO_2_ slabs with La atoms at the A site in the perovskite-type lattice. They introduced dopants into their model by replacing Fe atoms that were originally located at the B sites with the doping metal. The system was classified as exhibiting exsolution based on the location of the Fe atoms that were substituted into the lattice. Exsolution was predicted when the dopant was located at the uppermost layer; metal ions were assumed to remain in the lattice when the dopant occupied sites at or below the third FeO_2_ slab. Vacancies in the model were created by removing specific ions from the lattice.

Energy levels in the model system were calculated by considering all interactions among the various cations, anions and vacancies. The reducing atmosphere was considered to affect the energy levels of the crystal by changing the chemical potential of the oxygen ions. Exsolution in the model is considered thermodynamically favorable if the energy level of the “exsolution” case is lower than that of the “dissolution” case. Results from this model qualitatively agree with the experiments performed by Tanaka et al. [10], who found that exsolution of precious metals from LaFeO_3_ is facilitated in the order of Pd > Pt > Rh.

The model also revealed that oxygen vacancies in the lattice significantly stabilize Pd and Pt atoms on the surface. For instance, in the case of Pd, the “exsolution” state is slightly more stable than the “dissolution” state by 0.06 eV in a perfect LaFeO_3_ lattice, and this energy difference expanded to 0.88 eV after the introduction of oxygen vacancy. However, Rh exsolution was predicted to be energetically unfavorable in both “perfect” and “defective” cases.

DFT methods are very useful for describing systems where the initial and final states are well-known. The limitations of DFT methods for exsolution are also obvious in that the scale of DFT the model is often too small for accurate description of large particles. This, together with a paucity of quantitative experimental data on the process, has limited our ability to use the theories to make predictions for future studies. Progress is being made in this area but more work is required [75].

## 6. Properties of the Exsolved Particles

**Figure 5:**
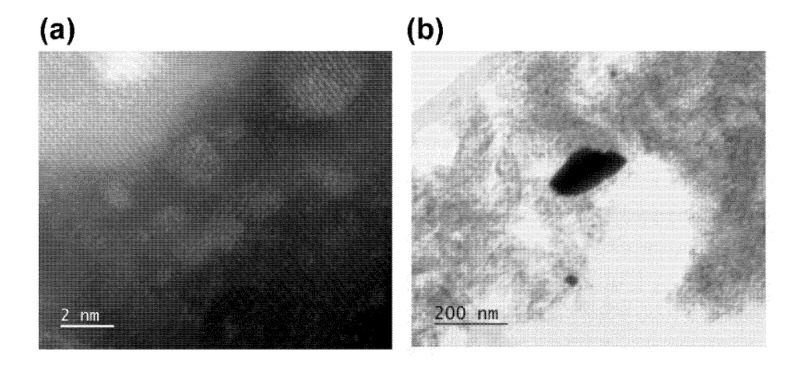
(**a**) High-angle annular dark-field STEM image of the reduced Pt exsolved from LaFeO_3_ thin film on MgAl_2_O_4_ support after 5 redox cycles at 800 °C; (**b**) high angle annular bright field STEM image of the reduced free standing Pt on MgAl_2_O_4_ support after 5 redox cycles at 800 °C; figures reprinted with permission from Ref. [77]. Copyright (2020) American Chemical Society.

The initial goal behind the development of “intelligent” catalysts was to regenerate catalysts that had undergone deactivation due to loss of dispersion caused by sintering of the metal [10]. The hypothesis was that metal cations could be reinserted into the lattice under oxidizing conditions and exsolved back as well-dispersed metals upon reduction. As discussed earlier [76], reinsertion of metal cations into the lattice appears to occur too slowly for this mechanism to be operative under practical conditions. Still, perovskite-supported metals do appear to maintain their dispersion much better than their conventional counterparts. This is shown by the example of Pt being supported on LaFeO_3_ films, supported on MgAl_2_O_4_, shown in Figure 5 [77]. Even after five redox cycles at 800 °C, the Pt particles on the LaFeO_3_ support existed as 1 to 2 nm particles, while Pt on MgAl_2_O_4_ agglomerated into large particles ranging from 20 to 100 nm after this pretreatment [77].

In at least some cases, the stabilization of metal dispersion can be explained by the socketed structure shown in Figure 4. Indeed, in a study of Ni exsolving from La_0.52_Sr_0.28_Ni_0.06_Ti_0.94_O_3−δ_ [11], the Ni particles were found to remain in fixed positions on the surface, even after very harsh treatments. Removing the particles by etching revealed pits in the support where the particles had been. However, there are cases where stabilization can only be explained by strong interactions between the metal and the support. For example, the stabilization in Figure 5 occurred with LaFeO_3_ films that were only 0.5 nm thick. This would appear to be too thin to allow socketing of the metals. The metal–support interactions are dependent on the compositions of both the metal [78] and the perovskite [79], suggesting that the nature of these interactions is similar to chemical bonds.

### Catalytic Properties

As discussed in the Introduction, the formation of carbon whiskers on Ni can lead to failure in SOFC Ni/YSZ cermet anodes when using hydrocarbon fuels. Carbon filament growth on Ni is reasonably well understood from work on methane-steam-reforming catalysis [80,81] and proceeds via a “tip growth” mechanism in which carbon deposited on the onto the surface of Ni particle dissolves into the metal and then precipitates as carbon fibers at an opposing surface or grain boundary [82]. Carbon fibers formed in this manner can lift the Ni particle off its support, resulting in the disintegration of the catalyst in a process that is known as metal dusting. Interestingly, it has been demonstrated that exsolved Ni particles do not catalyze the formation of carbon filaments [11,42]. The work of Neagu et al. [11] provides a good demonstration of the differences in the coking properties of exsolved and infiltrated Ni catalysts. Comparing Ni particles of a similar size, fewer carbon fibers are formed on exsolved Ni particles than formed on Ni particles prepared by infiltration on the same support. Apart from the difference in the propensity of the particles to catalyze the formation of carbon fibers, the manner of fiber growth also changed dramatically. The few carbon fibers that formed on the exsolved Ni grew along the surface of the oxide, rather than growing up-right and “lifting-off”. Similar coke tolerance was reported for Ni catalysts supported on from CaTiO_3_ [73,83]. 

The reasons for the coke tolerance in these perovskite-supported catalysts have yet to be fully explained. Because the exsolved Ni particles form a unique interface with the underlying host oxide, either through socketing or other strong metal–support interactions, it seems likely that nucleation of the carbon fibers is modified or somehow impeded, hindering carbon precipitation at the metal–oxide interface. Whether the formation of carbon in the metal particle is also impeded is not known.

Exsolved metals can also exhibit modified adsorption and reaction properties. For example, chemisorption of CO on Pt exsolved from LaFeO_3_ has been found to be negligible at room temperature, in spite of the fact that the catalytic activity towards CO oxidation is very high [77]. This suggests that the CO oxidation reaction on the exsolved catalysts is significantly different from that which takes place on catalysts prepared by conventional methods. Suppression of CO chemisorption has been observed on a wide range of perovskite-supported metals, including Pt on CaTiO_3_ [78] and LaCoO_3_ [84], Ni on LaFeO_3_ [73] and CaTiO_3_ [83], and Rh on ATiO_3_ (A=Ca, Sr and Ba) [79]. In the case of Pt on CaTiO_3_, the catalyst that was highly active for CO oxidation showed very low activities for hydrogenation reactions [78]. Possible explanations for the changes in chemisorption and reaction properties are partial covering of the metal with an oxide film and strong metal–support bonding interactions that modify the chemisorption properties of the metal. 

**Figure 6:**
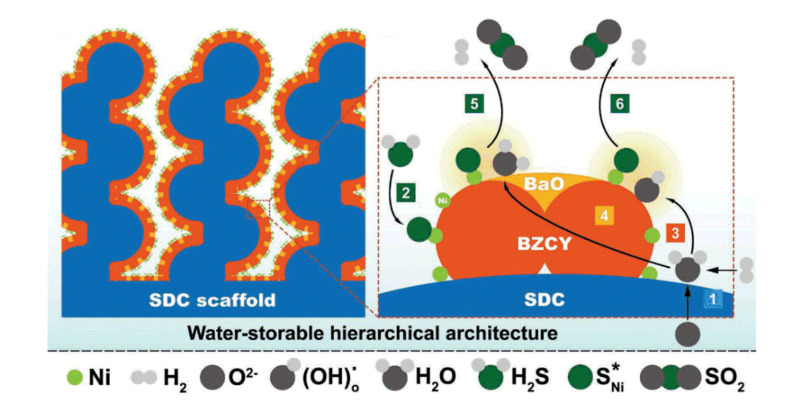
Proposed mechanism for water-induced sulfur removal from Ni decorated proton conducting perovskites (BaZr_0.32_Ce_0.32_Y_0.16_Ni_0.2_O_3−δ_), figure reprinted from Ref. [88].

There are also indications that exsolved metals may exhibit improved sulfur tolerance. Conventional Ni-based SOFC electrodes deactivate in the presence of H_2_S or other sulfur containing compounds because chemisorbed sulfur is very stable on the Ni surface [85,86]. H_2_S levels as low as 0.5 ppm can cause observable performance degradation of Ni-based anodes [87]. There are indications that sulfur adsorption is suppressed in exsolved Ni, thus allowing stable SOFC performance at higher sulfur levels. For example, Song et al. [88] studied an anode based on Ni exsolved from BaZr_0.4_Ce_0.4_Y_0.2_O_3−δ_ (BZCY) and found the anode provided stable operation for over 50 h when operating on a fuel which contained 100 ppm H_2_S. The authors reported that a similar cell with infiltrated Ni faded quickly under the same test conditions. The authors suggested two possibilities for the stability of the exsolved Ni anode. First, the strong interaction between Ni and the perovskite may have changed the electronic structure of Ni^0^. Alternatively, they suggested that dissociative adsorption of H_2_O on the BZCY may oxidize sulfur on neighboring Ni sites. The proposed mechanism of this sulfur removal process is presented in Figure 6.

Utilization of conductive ceramics in SOFC anodes, combined with the introduction of catalytic sites through exsolution, also allows the use of other metals [89] for the catalytic sites and some of these metals could be sulfur tolerant. For example, Cui et al. [90] incorporated Co into lanthanum titanate (LSCoT, La_0.3_Sr_0.7_Co_0.07_Ti_0.93_O_3−δ_) to achieve a cobalt-based exsolution anode material. Although cobalt and nickel have similar affinities for sulfur [91], the authors reported the anode based on exsolved Co was stable for 48 h operating in 5000 ppm H_2_S.

## 7. Summary

In this review, we have attempted to show that exsolution is a widely applicable approach to adding catalysts to SOFC anodes. To demonstrate that, a list of some of the materials that have been prepared by this approach is presented in Table 1. This list is not complete, given that new materials are being reported weekly in the literature. The list includes the chemical formula of the host oxide, the metal exsolved, the characteristic metal-particle size and cell configuration. The structure of the host (e.g., “simple” perovskite, Ruddlesden–Popper (RP) perovskite and layered double perovskite) is also included.

## 8. Outlook

In this brief review, we have attempted to highlight some of the interesting properties of SOFC anodes that use perovskite-type oxides for electronic conductivity and exsolved metals to enhance catalytic activity. As the examples demonstrate, exsolved metals form unique interfaces with their perovskite hosts, in some cases becoming socketed into the surfaces of the perovskite and forming chemical bonds in other cases. These strong interactions between the metal and the perovskite surface serve to both stabilize the metal from coarsening at high temperatures and, in the case of Ni, disrupt the mechanism by which metal particles catalyze the formation of carbon filaments when exposed to hydrocarbons. In some cases, the exsolved metals also appear to have high sulfur tolerance which is not observed for supported metals formed by more conventional means. The properties of exsolution catalysts make these materials extremely promising for use in SOFC anodes.

However, while perovskite-based anodes with exsolved metal catalysts have much promise, there is still great need for a fundamental understanding of the exsolution process and how to best use these materials. The compositional specificity observed in these systems implies that every metal/perovskite combination is likely to have unique properties. Thus, there is still much need for research in this area to understand the physics of the exsolution and the factors that control the structure and catalytic activity of the exsolved metals. A better understanding of these fundamental questions may pave the way for the use of this concept in highly stable and active SOFC anodes.

## Figures and Tables

**Table 1:** Exsolution Materials developed for Solid Oxide Cells (SOCs).

Pristine Phase	Operation Mode	Metal	Hosting Phase	Particle Parameters	Cell Configuration
BaCe_0.72_Y_0.08_Ni_0.2_O_3−δ_ [92]	SOFC	Ni	simple	--	BCYNi infiltrated GDC (Gd_0.1_Ce_0.9_O_1.95_)|GDC|LSCF (La_0.6_Sr_0.4_Co_0.2_Fe_0.8_O_3−δ_)-GDC
BaZr_0.32_Ce_0.32_Y_0.16_Ni_0.2_O_3−__δ_ [88]	SOFC	Ni	simple	~30 nm dia.	BZCYNi infiltrated SDC (Sm_0.2_Ce_0.8_O_1.9_)|SDC|BSCF (Ba_0.5_Sr_0.5_Co_0.8_Fe_0.2_O_3−δ_) infiltrated SDC
Ce_1__−__x_Ni_x_VO_4__−δ_ family and Ce_0.7_(Sr/Ca)_0.1_Ni_0.2_VO_4__−δ_ [12]	SOFC	Ni	simple	10–20 nm dia.	doped vanadate-YSZ|YSZ|LSF (La_0.8_Sr_0.2_FeO_3_)-YSZ
La_0.6_Ca_0.4_Fe_0.8_Ni_0.2_O_3−__δ_ [93]	SOFC	Ni	simple	~10 nm	LCFNi infiltrated SDC (Sm_0.2_Ce_0.8_O_1.9_)|SDC|LCFNi infiltrated SDC symmetric cell
La_0.3_Sr_0.6_Ce_0.1_Ni_0.1_Ti_0.9_O_3−δ_ and La_0.4_Sr_0.6_Ni_0.1_Ti_0.9_O_3−δ_ [94]	SOFC	Ni	simple	~20 nm dia.	LSCNT/LSNT|YSZ|LSM-YSZ
La_0.675_Sr_0.225_Cr_0.45_Mn_0.45_Ni_0.1−x_Cu_x_O_3−__δ_ family [95]	SOEC	Ni-Cu	simple	~40 nm dia.	LSCMNiCu-SDC (Ce_0.8_Sm_0.2_O_2−δ_)|LSGM (La_0.9_Sr_0.1_Ga_0.8_Mg_0.2_O_3_)|LSM (La_0.76_Sr_0.19_MnO_3−δ_)-SDC
La_0.8_Sr_0.2_Cr_1-x_Ni_x_O_3−δ_ [96]	SOFC	Ni	simple	15–20 nm dia.	LSCrNi-GDC (Ce_0.9_Gd_0.1_O_1.95_)|LSGM (La_0.9_Sr_0.1_Ga_0.8_Mg_0.2_O_3−__δ_)|LSCF (La_0.6_Sr_0.4_Co_0.2_Fe_0.8_O_3−__δ_)-GDC
La_0.4_Sr_0.6_Fe_0.75_Ni_0.1_Nb_0.1_5O_3−δ_ [38]	SOFC	Fe-Ni	RP-simple	20–40 nm dia.	LSFeNiN|SDC (Sm_0.2_Ce_0.8_O_1.9_)|ScCeSZ ((Sc_0.2_O_0.3_)_0.1_(CeO_2_)_0.01_(ZrO_2_)_0.89_)|SDC|LSFCN (La_0.5_Sr_0.5_Fe_0.8_Cu_0.15_Nb_0.05_O_3−δ_)
La_0.4_Sr_0.4_Ni_0.06_Ti_0.94_O_3−δ_ [35]	SOEC	Ni	simple	60–90 nm dia.	LSNiT|YSZ|LSM (La_0.76_Sr_0.19_MnO_3_)-YSZ
La_0.75_Sr_0.25_Cr_0.5_Mn_0.3_Ni_0.2_O_3−δ_ [97]	SOFC	Ni	simple	~15 nm dia.	LSCMNi|Ce_0.9_Gd_0.1_O_1.95_ (GDC)|LSCMNi symmetric cell for impedance testing
La_0.8_Sr_0.2_Cr_0.69_Ni_0.31_O_3−δ_ family [98]	SOFC	Ni	simple	10–50 nm dia.	LSCrNi-GDC|LSGM (La_0.9_Sr_0.1_Ga_0.8_Mg_0.2_O_3−δ_)|LSCF (La_0.6_Sr_0.4_Co_0.2_Fe_0.8_O_3−δ_)-GDC
La_0.85_Sr_0.15_Cr_1__−__y_Ni_y_O_3−__δ_ family [99]	SOFC	Ni	simple	15–20 nm dia.	LSCrN|YSZ|LSCrN symmetric cell for impedance testing
La_0.7_Sr_0.3_Cr_0.85_Ni_0.15_O_3−δ_ and La_0.7_Sr_0.3_Cr_0.85_Ni_0.1125_Fe_0.0375_O_3−δ_ [100]	SOFC	Ni/Ni-Fe	simple	25–30 nm dia.	LSCN/LSCNF-YSZ|YSZ|LSM-YSZ
LaSrFeNiO_6__−δ_ [101]	SOFC	Ni-Fe	RP	30–50 nm dia.	LSFeNi infiltrated SDC (Sm_0.2_Ce_0.8_O_1.9_)|SDC|SSC (Sm_0.5_Sr_0.5_CoO_3−δ_) infiltrated SDC
La_0.5_Sr_0.5_Fe_0.8_Ni_0.2_O_3−δ_ [102]	SOFC	Ni-Fe	RP	~25 nm dia.	LSFN|SDC (Sm_0.2_Ce_0.8_O_1.9_)|ScCeSZ ((Sc_0.2_O_0.3_)_0.1_(CeO_2_)_0.01_(ZrO_2_)_0.89_)|SDC|LSFCN (La_0.5_Sr_0.5_Fe_0.8_Cu_0.15_Nb_0.05_O_3−δ_)
La_0.4_Sr_0.4_Sc_0.9_Ni_0.1_O_3−δ_ [13]	SOFC	Ni	simple	~50 nm dia.	LSSN-SDC (samarium-doped ceria)|SDC|LSSN-SDC symmetric cell for impedance testing
La_0.2_Sr_0.8_Ti_1__−__x_Ni_x_O_3−__δ_ [103]	SOFC	Ni	simple	~7 nm dia.	LSTNi|(ScCeSZ) ((Sc_2_O_3_)_0.1_(CeO_2_)_0.01_(ZrO_2_)_0.89_)|Gd_0.2_Ce_0.8_O_2_ (GDC)|La_0.6_Sr_0.4_Co_0.2_Fe_0.8_O_3_ (LSCF)-Gd_0.1_Ce_0.9_O_2_ (GDC) electrolyte-supported
La_0.2_Sr_0.8_Ti_0.9_Ni_0.1_O_3−δ_ [104]	SOFC	Ni	simple	~7 nm dia.	LSTNi|(Sc_2_O_3_)_0.1_(CeO_2_)_0.01_(ZrO_2_)_0.89_ (ScCeSZ)|Gd_0.2_Ce_0.8_O_2_ (GDC)|La_0.6_Sr_0.4_Co_0.2_Fe_0.8_O_3_ (LSCF)-Gd_0.1_Ce_0.9_O_2_ (GDC) electrolyte-supported
La_0.5_Sr_0.5_Ti_0.75_Ni_0.25_O_3−__δ_ [105]	SOFC	Ni	simple	5–50 nm dia.	LSTNi|yttria-doped ceria (YDC)|YSZ|YDC|LSTNi symmetric cell for impedance testing
La_0.19_Sr_0.76_Ti_0.85_Cr_0.1_Ni_0.05_O_3+__δ_/La_0.19_Sr_0.76_Ti_0.85_Mn_0.1_Ni_0.05_O_3+__δ_ [106]	SOEC	Ni	simple	~60 nm dia.	LSTCNi/LSTMNi-SDC (Ce_0.8_Sm_0.2_O_2-δ_)|YSZ|LSM (La_0.76_Sr_0.19_MnO_3−δ_)-YSZ
NbTi_0.5_Ni_0.5_O_4_ [59]	SOFC	Ni	rutile	~80 nm dia.	NTNO-YSZ|YSZ|NTNO-YSZ symmetric cell for impedance tests
Pr_0.65_Ba_0.35_Mn_0.975_Ni_0.025_O_3_ [107]	SOFC	Ni	layered double	~80 nm dia.	PBMNi|Ce_0.9_Gd_0.1_O_1.95_ (GDC)|YSZ|GDC|PBMNi symmetric cell for impedance tests
PrBaMn_1.7_Co_0.1_Ni_0.2_O_5+__δ_ [108]	SOFC	Co-Ni	layered double	~40 nm dia.	PBMCNO|La_0.4_Ce_0.6_O_2−δ_ (LDC)|La_0.9_Sr_0.1_Ga_0.8_Mg_0.2_O_3−δ_ (LSGM)|NdBa_0.5_Sr_0.5_Co_1.5_Fe_0.5_O_5+δ_–Ce_0.9_Gd_0.1_O_2-δ_ (NBSCF50-GDC)
Pr_0.5_Ba_0.5_Mn_0.85_(Ni/Co)_0.15_O_3−δ_ [36]	SOFC	Ni, Co, respectively	layered double	20–50 nm dia.	PBMNi/Co|LDC (La_0.4_Ce_0.6_O_2-δ_)|LSGM (La_0.9_Sr_0.1_Ga_0.8_Mg_0.2_O_3−δ_)|NdBSCF (NdBa_0.5_Sr_0.5_Co_1.5_Fe_0.5_O_5-δ_)-GDC (Ce_0.9_Gd_0.1_O_2−δ_)
Sr_2_FeMo_1−x_Ni_x_O_6−δ_ family [52]	SOFC	Fe-Ni	RP and simple	50–60 nm dia.	SFMNi|La_0.4_Ce_0.6_O_2__−δ_(LDC)|La_0.8_Sr_0.2_Ga_0.87_Mg_0.13_O_3_ (LSGM)|La_0.58_Sr_0.4_Co_0.2_Fe_0.8_O_3−__δ_ (LSCF)
Sr_2_Fe_1.3_Mo_0.5_Ni_0.2_O_6−__δ_ [109]	SOEC	Fe-Ni	simple	~30 nm dia., ~500 μm^−2^	SFMNi–Sm_0.2_Ce_0.8_O_1.9_ (SDC)|La_0.5_Ce_0.5_O_1.5_ (LDC)|La_0.8_Sr_0.2_Ga_0.87_Mg_0.13_O_3_ (LSGM)|SDC–La_0.6_Sr_0.4_Co_0.2_Fe_0.8_O_3−__δ_ (LSCF)
Sr_0.95_Ti_0.3_Fe_0.63_Ni_0.07_O_3−__δ_ [68], SrTi_0.3_Fe_0.63_Ni_0.07_O_3−__δ_ [67]	SOFC	Fe-Ni	simple	STFN: 40–70 nm dia. ~240 μm^−2^ S95TFN: 20–70 nm dia. ~210 μm^−2^	STFeNi|LDC (La_0.4_Ce_0.6_O_2−δ_)|LSGM (La_0.8_Sr_0.2_Ga_0.83_Mg_0.17_O_3−δ_)|LSCF (La_0.6_Sr_0.4_Co_0.2_Fe_0.8_O3_3−δ_)-GDC (Gd_0.1_Ce_0.9_O_1.95_)
Sr_0.95_Ti_0.76_Nb_0.19_Ni_0.05_O_3−δ_ [110]	SOFC	Ni	simple	~50 nm dia.	STNNi- Ce_0.8_Sm_0.2_O_2_ (SDC)|La_0.4_Ce_0.6_O_2_ (LDC)|La_0.8_Sr_0.2_Ga_0.87_Mg_0.13_O_3_ (LSGM)|La_0.6_Sr_0.4_Co_0.2_Fe_0.8_O_3_ (LSCF)
La_0.43_Ca_0.37_Rh_0.06_Ti_0.94_O_3_ [111]	SOEC	Rh	simple	2.1–3.2 nm dia.; 11,000–5100 μm^−2^	LCRhT|GDC|ScCeSZ|GDC|LCRhT
La_0.75_Sr_0.25_Cr_0.50−x_Mn_0.50-y_Ru_x,y_O_3_ family [112]	SOFC	Ru	simple	5–10 nm dia.	LSCMRu|Ni-YSZ|YSZ|La_0.65_Sr_0.3_MnO_3_ (LSM)
La_0.8_Sr_0.2_Cr_0.82_Ru_0.18_O_3−δ_ [98,113]	SOFC	Ru	simple	~2.9 nm	LSCrRu-GDC (Ce_0.9_Gd_0.1_O_1.95_)|LSGM (La_0.9_Sr_0.1_Ga_0.8_Mg_0.2_O_3−δ_)|LSCF (La_0.6_Sr_0.4_Co_0.2_Fe_0.8_O_3−δ_)-GDC
La_0.8_Sr_0.2_Cr_1__−__x_Ru_x_O_3−δ_ family [14,96,114]	SOFC	Ru	simple	≤5 nm dia.	LSCrRu-GDC (Ce_0.9_Gd_0.1_O_1.95_)|LSGM (La_0.9_Sr_0.1_Ga_0.8_Mg_0.2_O_3−δ_)|LSCF (La_0.6_Sr_0.4_Co_0.2_Fe_0.8_O_3−δ_)-GDC
La_0.8_Sr_0.2_Cr_0.8_Pd_0.2_O_3−δ_, La_0.8_Sr_0.2_Cr_0.95_Pd_0.05_O_3−__δ_ [115]	SOFC	Pd	simple	~8 nm dia.	LSCPd-GDC (Ce_0.9_Gd_0.1_O_2−__δ_)|LSGM (La_0.9_Sr_0.1_Ga_0.8_Mg_0.2_O_3−__δ_)|LSCF (La_0.6_Sr_0.4_Fe_0.8_Co_0.2_O_3−__δ_)-GDC
La_0.6_Sr_0.4_Fe_0.95_Pd_0.05_O_3−δ_ [116]	SOFC	Pd and Fe separately	RP and simple	10–15 nm dia.	LSFPd-Ce_0.9_Gd_0.1_O_2_ (GDC)|La_0.8_Sr_0.2_Ga_0.8_Mg_0.2_O_3−δ_ (LSGM)|LSFPd
La_0.6_Sr_0.4_Fe_0.85_Pd_0.05_Mn_0.9_O_3_ [117]	SOFC	Pd	simple	~10 nm dia.	LSFMP|La_0.8_Sr_0.2_Ga_0.8_Mg_0.15_Co_0.05_O_3_ (LSGMC)|Sm_0.5_Sr_0.5_CoO_3_ (SSC) electrolyte-supported
SrTi_0.3_Fe_0.7_Ru_0.07_O_3−δ_ [118]	SOFC	Ru-Fe	simple	5–20 nm dia.	STFRu|LDC (La_0.4_Ce_0.6_O_2_)|LSGM (La_0.8_Sr_0.2_Ga_0.83_Mg_0.17_O_3_)|LSCF (La_0.6_Sr_0.4_Fe_0.8_Co_0.2_O_3_)-GDC (Ce_0.9_Gd_0.1_O_2_)
Ce_1−x_(Co/Cu)_x_VO_4−δ_ families [12]	SOFC	Co, Cu	simple	10–20 nm dia.	CeTMV-YSZ|YSZ|LSF (La_0.8_Sr_0.2_FeO_3_)-YSZ
Ce_0.8_Sr_0.1_Cu_0.05_Co_0.05_VO_4-δ_ [31]	SOFC	Cu, Co mixture with separate phases	simple	10–20 nm dia.	CeSrCuCV-YSZ|YSZ|LSF (La_0.8_Sr_0.2_FeO_3_)-YSZ
La_0.43_Ca_0.37_Ni_0.06_Ti_0.94_O_3−δ_/La_0.43_Ca_0.37_Ni_0.03_Fe_0.03_Ti_0.94_O_3−δ_ [119]	SOFC and SOEC	Ni/Ni-Fe alloy	simple	30–100 nm dia.	LCNiT/LCNiFeT|ScCeSZ|LSM (La_0.76_Sr_0.19_MnO_3_)-ScCeSZ
La_0.3_Sr_0.7_Co_0.07_Ti_0.9_3O_4__−δ_ [90]	SOFC	Co	simple	~5 nm dia.	LSCT-YSZ|YSZ|LSM-YSZ
La_0.7_Sr_0.3_Cr_0.85_Fe_0.15_O_3−δ_, La_0.6_Sr_0.3_Cr_0.85_Fe_0.15_O_3−δ_ [120]	SOFC	Fe	simple	~25 nm dia.	LSCrF-YSZ|YSZ|LSM-YSZ
La_0.5_Sr_1.5_Fe_1.5_Mo_0.5_O_6-__δ_ [121,122]	SOFC	Fe	RP and simple	~100 nm dia.	LSFM|LSGM|LSFM symmetric cell for impedance testing
La_0.675_Sr_0.225_Cr_0.45_Mn_0.45_Cu_0.1-x_Fe_x_O_3−δ_ family [123]	SOFC	Cu-Fe	simple	~30 nm dia.	LSCMCuFe-SDC (Ce_0.8_Sm_0.2_O_2-δ_)|LDC (Ce_0.6_La_0.4_O_2_)|LSGM (La_0.9_Sr_0.1_Ga_0.8_Mg_0.2_O_3−δ_)|LSM (La_0.8_Sr_0.2_MnO_3−δ_)-SDC
La_0.5_Sr_0.5_Fe_0.8_Cu_0.15_Nb_0.05_O_3−δ_ [19]	SOFC	Cu	RP-simple	5–10 nm dia.	LSFCuN|SDC (Sm_0.2_Ce_0.8_O_1.9_)|ScCeSZ ((Sc_2_O_3_)_0.1_(CeO_2_)_0.01_(ZrO_2_)_0.89_)|SDC|LSFCuN
La_0.8_Sr_0.2_Fe_0.9_Nb_0.1_O_3−δ_ [124]	SOFC	Fe	simple	less than 50 nm dia.	LSFNb|Sm_0.2_Ce_0.8_O_2−δ_ (SDC)|(Sc_2_O_3_)_0.1_(CeO_2_)_0.01_(ZrO_2_)_0.89_ (ScCeSZ)|(La_0.75_Sr_0.25_)_0.95_MnO_3−δ_-ScCeSZ
La_0.5_Sr_0.5_Co_0.45_Fe_0.45_Nb_0.1_O_3−δ_ [125]	SOFC	Co-Fe	simple	30–50 nm dia.	LSCoFeN|LSGM (La_0.8_Sr_0.2_Ga_0.83_Mg_0.17_O_3−δ_)|LSCoFeN symmetric cell for impedance testing
La_0.3_Sr_0.7_Cr_0.3_Fe_0.6_Co_0.1_O_3−__δ_ [126]	SOFC	Co-Fe	simple	~30 nm dia.	LSCrFeCo-GDC|La_0.4_Ce_0.6_O_2−δ_ (LDC)|La_0.8_Sr_0.2_Ga_0.8_Mg_0.2_O_3−δ_ (LSGM)|LSCF-GDC
La_0.8_Sr_1.2_Fe_0.9_Co_0.1_O_4-__δ_ [50]	SOFC	Co	RP	~10 nm dia.	LSFC-LSGM (La_0.9_Sr_0.1_Ga_0.8_Mg_0.2_O_3−δ_)-GDC multilayered anode |LSGM|LSFC-LSGM-GDC symmetric cell for impedance testing
La_0.4_Sr_0.4_Fe_0.06_Ti_0.94_O_3−δ_ [35]	SOEC	Fe	simple	30–60 nm dia.	LSFeTi|YSZ|LSM (La_0.76_Sr_0.19_MnO_3_)-YSZ
Pr_0.5_Ba_0.5_Mn_0.9_Co_0.1_O_3−δ_ [48]	SOEC/SOFC	Co	layered double	20–30 nm dia.	PBMCo-GDC|YSZ|LSM-DGC
Pr_0.4_Sr_0.6_Co_0.2_Fe_0.7_Nb_0.1_O_3−δ_ [127]	SOFC	Co-Fe	RP	~30 nm dia.	PSCFN|LDC (La_0.4_Ce_0.6_O_2-__δ_)|LSGM (La_0.8_Sr_0.2_Ga_0.83_Mg_0.17_O_3−δ_)|BCFN (Ba_0.9_Co_0.7_Fe_0.2_Nb_0.1_O_3−δ_)
Pr_0.4_Sr_0.6_Co_0.2_Fe_0.7_Nb_0.1_O_3−δ_ [128]	SOFC	Co-Fe	RP	~50 nm dia.	PSCoFeN|LSGM (La_0.8_Sr_0.2_Ga_0.83_Mg_0.17_O_3−δ_)|PSCoFeN, PSCFN|LSGM|BCFN (Ba_0.9_Co_0.7_Fe_0.2_Nb_0.1_O_3−δ_) and PSCoFeN|YSZ|BCFN
Pr_0.4_Sr_0.6_Co_0.2_Fe_0.7_Mo_0.1_O_3−δ_ [51]	SOEC	Co-Fe	RP	~50 nm dia.	PSCFM-GDC (Gd_0.2_Ce_0.8_O_2-δ_)|GDC|YSZ|GDC|LSCF (La_0.58_Sr_0.38_Co_0.20_Fe_0.80_O_3−δ_)-GDC
Sr_2_Fe_1.3_Co_0.2_Mo_0.5_O_6−__δ_ [129]	SOFC	Co	RP	~50 nm dia.	SFCM|LSGM (La_0.8_Sr_0.2_Ga_0.83_Mg_0.17_O_3−δ_)|LSCF (La_0.6_Sr_0.4_Co_0.2_Fe_0.8_O_3−δ_)
Sr_2_Fe_1.35_Mo_0.45_Co_0.2_O_6−δ_ [56]	SOEC	Co-Fe	RP and double	exsolved under H_2_ at 850 °C for different length of time. 1 h: ~12 nm, ~750 μm^−2^; 2 h: ~18 nm, ~780 μm^−2^; 4 h: ~25 nm; 680 μm^−2^	LCRhT|GDC|ScCeSZ|GDC|LCRhT
Sr_2_FeMo_0.67_Co_0.33_O_6−δ_ [130]	SOFC	Co-Fe	RP	10–50 nm	SFMCo-SDC (Sm_0.2_Ce_0__.8_O_1.9_)|LDC (La_0__.4_Ce_0__.6_O_2−δ_)|LSGM (La_0__.8_Sr_0.2_Ga_0.8_Mg_0__.2_O_3−__δ_)|LSCF (La_0__.58_Sr_0__.4_Co_0__.2_Fe_0__.8_O_3−__δ_)-SDC

## References

[1] Minh N.Q. (1993). Ceramic fuel cells. J. Am. Ceram. Soc..

[2] Steele B.C., Heinzel A. (2001). Materials for fuel-cell technologies. Nature.

[3] Pihlatie M., Ramos T., Kaiser A. (2009). Testing and improving the redox stability of Ni-based solid oxide fuel cells. J. Power Sources.

[4] Hanna J., Lee W.Y., Shi Y., Ghoniem A. (2014). Fundamentals of electro-and thermochemistry in the anode of solid-oxide fuel cells with hydrocarbon and syngas fuels. Prog. Energy Combust. Sci..

[5] He H., Gorte R.J., Vohs J.M. (2005). Highly sulfur tolerant Cu-ceria anodes for SOFCs. Electrochem. Solid-State Lett..

[6] Ahn K., Jung S., Vohs J.M., Gorte R.J. (2007). A support layer for solid oxide fuel cells. Ceram. Int..

[7] Tucker M.C., Lau G.Y., Jacobson C.P., DeJonghe L.C., Visco S.J. (2007). Performance of metal-supported SOFCs with infiltrated electrodes. J. Power Sources.

[8] Kim G., Lee S., Shin J., Corre G., Irvine J., Vohs J., Gorte R.J. (2009). Investigation of the structural and catalytic requirements for high-performance SOFC anodes formed by infiltration of LSCM. Electrochem. Solid State Lett..

[9] Simwonis D., Tietz F., Stöver D. (2000). Nickel coarsening in annealed Ni/8YSZ anode substrates for solid oxide fuel cells. Solid State Ion..

[10] Tanaka H., Taniguchi M., Uenishi M., Kajita N., Tan I., Nishihata Y., Mizuki J.I., Narita K., Kimura M., Kaneko K. (2006). Self-regenerating Rh-and Pt-based perovskite catalysts for automotive-emissions control. Angewante Chem. Int. Ed..

[11] Neagu D., Oh T.-S., Miller D.N., Ménard H., Bukhari S.M., Gamble S.R., Gorte R.J., Vohs J.M., Irvine J.T. (2015). Nano-socketed nickel particles with enhanced coking resistance grown in situ by redox exsolution. Nat. Commun..

[12] Adijanto L., Balaji Padmanabhan V., Küngas R., Gorte R.J., Vohs J.M. (2012). Transition metal-doped rare earth vanadates: A regenerable catalytic material for SOFC anodes. J. Mater. Chem..

[13] Gao Y., Chen D., Saccoccio M., Lu Z., Ciucci F. (2016). From material design to mechanism study: Nanoscale Ni exsolution on a highly active A-site deficient anode material for solid oxide fuel cells. Nano Energy.

[14] Madsen B.D., Kobsiriphat W., Wang Y., Marks L.D., Barnett S.A. (2007). Nucleation of nanometer-scale electrocatalyst particles in solid oxide fuel cell anodes. J. Power Sources.

[15] Tanner C.W., Fung K.Z., Virkar A.V. (1997). The effect of porous composite electrode structure on solid oxide fuel cell performance: I. theoretical analysis. J. Electrochem. Soc..

[16] Vohs J.M., Gorte R.J. (2009). High-performance SOFC cathodes prepared by infiltration. Adv. Mater..

[17] Gorte R.J., Park S., Vohs J.M., Wang C. (2000). Anodes for direct oxidation of dry hydrocarbons in a solid-oxide fuel cell. Adv. Mater..

[18] Kim H., Park S., Vohs J.M., Gorte R.J. (2001). Direct oxidation of liquid fuels in a solid oxide fuel cell. J. Electrochem. Soc..

[19] Zhou N., Yin Y.-M., Chen Z., Song Y., Yin J., Zhou D., Ma Z.-F. (2018). A regenerative coking and sulfur resistant composite anode with Cu exsolution for intermediate temperature solid oxide fuel cells. J. Electrochem. Soc..

[20] Jung S., Lu C., He H., Ahn K., Gorte R.J., Vohs J.M. (2006). Influence of composition and Cu impregnation method on the performance of Cu/CeO2/YSZ SOFC anodes. J. Power Sources.

[21] Gasper P., Lu Y., Basu S.N., Gopalan S., Pal U.B. (2019). Effect of anodic current density on the spreading of infiltrated nickel nanoparticles in nickel-yttria stabilized zirconia cermet anodes. J. Power Sources.

[22] Corre G., Kim G., Cassidy M., Vohs J., Gorte R., Irvine J. (2009). Activation and ripening of impregnated manganese containing perovskite SOFC electrodes under redox cycling. Chem. Mater..

[23] Adijanto L., Sampath A., Yu A.S., Cargnello M., Fornasiero P., Gorte R.J., Vohs J.M. (2013). Synthesis and Stability of Pd@CeO_2_ Core–Shell Catalyst Films in Solid Oxide Fuel Cell Anodes. ACS Catal..

[24] Graham G., Jen H.-W., Chun W., Sun H., Pan X., McCabe R. (2004). Coarsening of Pt particles in a model NO x trap. Catal. Lett..

[25] Hansen T.W., DeLaRiva A.T., Challa S.R., Datye A.K. (2013). Sintering of catalytic nanoparticles: Particle migration or Ostwald ripening?. Acc. Chem. Res..

[26] He J.-J., Wang C.-X., Zheng T.-T., Zhao Y.-K. (2016). Thermally induced deactivation and the corresponding strategies for improving durability in automotive three-way catalysts. Johns. Matthey Technol. Rev..

[27] Tanaka H., Tan I., Uenishi M., Kimura M., Dohmae K. (2001). Regeneration of palladium subsequent to solid solution and segregation in a perovskite catalyst: An intelligent catalyst. Top. Catal..

[28] Nishihata Y., Mizuki J., Akao T., Tanaka H., Uenishi M., Kimura M., Okamoto T., Hamada N. (2002). Self-regeneration of a Pd-perovskite catalyst for automotive emissions control. Nature.

[29] Tanaka H., Taniguchi M., Kajita N., Uenishi M., Tan I., Sato N., Narita K., Kimura M. (2004). Design of the intelligent catalyst for Japan ULEV standard. Top. Catal..

[30] Uenishi M., Taniguchi M., Tanaka H., Kimura M., Nishihata Y., Mizuki J., Kobayashi T. (2005). Redox behavior of palladium at start-up in the Perovskite-type LaFePdOx automotive catalysts showing a self-regenerative function. Appl. Catal. B Environ..

[31] Adijanto L., Padmanabhan V.B., Gorte R.J., Vohs J.M. (2012). Polarization-Induced Hysteresis in CuCo-Doped Rare Earth Vanadates SOFC Anodes. J. Electrochem. Soc..

[32] Myung J.H., Neagu D., Miller D.N., Irvine J.T. (2016). Switching on electrocatalytic activity in solid oxide cells. Nature.

[33] Opitz A.K., Nenning A., Rameshan C., Rameshan R., Blume R., Havecker M., Knop-Gericke A., Rupprechter G., Fleig J., Klotzer B. (2015). Enhancing electrochemical water-splitting kinetics by polarization-driven formation of near-surface iron(0): An in situ XPS study on perovskite-type electrodes. Angewante Chem. Int. Ed..

[34] Opitz A.K., Nenning A., Vonk V., Volkov S., Bertram F., Summerer H., Schwarz S., Steiger-Thirsfeld A., Bernardi J., Stierle A. (2020). Understanding electrochemical switchability of perovskite-type exsolution catalysts. Nat. Commun..

[35] Tsekouras G., Neagu D., Irvine J.T.S. (2013). Step-change in high temperature steam electrolysis performance of perovskite oxide cathodes with exsolution of B-site dopants. Energy Environ. Sci..

[36] Kwon O., Sengodan S., Kim K., Kim G., Jeong H.Y., Shin J., Ju Y.-W., Han J.W., Kim G. (2017). Exsolution trends and co-segregation aspects of self-grown catalyst nanoparticles in perovskites. Nat. Commun..

[37] Shen X., Chen T., Bishop S., Perry N.H., Tuller H., Sasaki K. (2017). Redox cycling induced Ni exsolution in Gd_0.1_Ce_0.8_Ni_0.1_O_2_-(Sr_0.9_La_0.1_)_0.9_Ti_0.9_Ni_0.1_O_3_ composite solid oxide fuel cell anodes. J. Power Sources.

[38] Li J., Yu Y., Yin Y.-M., Zhou N., Ma Z.-F. (2017). A novel high performance composite anode with in situ growth of Fe-Ni alloy nanoparticles for intermediate solid oxide fuel cells. Electrochim. Acta.

[39] Oemar U., Ang M., Hee W., Hidajat K., Kawi S. (2014). Perovskite La_x_M_1−x_Ni_0.8_Fe_0.2_O_3_ catalyst for steam reforming of toluene: Crucial role of alkaline earth metal at low steam condition. Appl. Catal. B Environ..

[40] Thalinger R., Gocyla M., Heggen M., Dunin-Borkowski R., Grünbacher M., Stöger-Pollach M., Schmidmair D., Klötzer B., Penner S. (2016). Ni–perovskite interaction and its structural and catalytic consequences in methane steam reforming and methanation reactions. J. Catal..

[41] Neagu D., Tsekouras G., Miller D.N., Ménard H., Irvine J.T. (2013). In situ growth of nanoparticles through control of non-stoichiometry. Nat. Chem..

[42] Oh T.-S., Rahani E.K., Neagu D., Irvine J.T., Shenoy V.B., Gorte R.J., Vohs J.M. (2015). Evidence and model for strain-driven release of metal nanocatalysts from perovskites during exsolution. J. Phys. Chem. Lett..

[43] Neagu D., Papaioannou E.I., Ramli W.K., Miller D.N., Murdoch B.J., Ménard H., Umar A., Barlow A.J., Cumpson P.J., Irvine J.T. (2017). Demonstration of chemistry at a point through restructuring and catalytic activation at anchored nanoparticles. Nat. Commun..

[44] Ge X.-M., Chan S.-H., Liu Q.-L., Sun Q. (2012). Solid Oxide Fuel Cell Anode Materials for Direct Hydrocarbon Utilization. Adv. Energy Mater..

[45] Dimitrakopoulos G., Ghoniem A.F., Yildiz B. (2019). In situ catalyst exsolution on perovskite oxides for the production of CO and synthesis gas in ceramic membrane reactors. Sustain. Energy Fuels.

[46] Steele B., Middleton P., Rudkin R. (1990). Material science aspects of SOFC technology with special reference to anode development. Solid State Ion..

[47] Tao S., Irvine J.T. (2003). A redox-stable efficient anode for solid-oxide fuel cells. Nat. Mater..

[48] Sun Y.F., Zhang Y.Q., Chen J., Li J.H., Zhu Y.T., Zeng Y.M., Amirkhiz B.S., Li J., Hua B., Luo J.L. (2016). New Opportunity for in Situ Exsolution of Metallic Nanoparticles on Perovskite Parent. Nano Lett..

[49] Kwon O., Joo S., Choi S., Sengodan S., Kim G. (2020). Review on exsolution and its driving forces in perovskites. J. Phys. Energy.

[50] Zhou J., Shin T.-H., Ni C., Chen G., Wu K., Cheng Y., Irvine J.T.S. (2016). In Situ Growth of Nanoparticles in Layered Perovskite La_0.8_Sr_1.2_Fe_0.9_Co_0.1_O_4−δ_ as an Active and Stable Electrode for Symmetrical Solid Oxide Fuel Cells. Chem. Mater..

[51] Liu S., Liu Q., Luo J.-L. (2016). CO2-to-CO conversion on layered perovskite with in situ exsolved Co–Fe alloy nanoparticles: An active and stable cathode for solid oxide electrolysis cells. J. Mater. Chem. A.

[52] Du Z., Zhao H., Yi S., Xia Q., Gong Y., Zhang Y., Cheng X., Li Y., Gu L., Swierczek K. (2016). High-Performance Anode Material Sr2FeMo0.65Ni0.35O6-delta with In Situ Exsolved Nanoparticle Catalyst. ACS Nano.

[53] Choi S., Sengodan S., Park S., Ju Y.W., Kim J., Hyodo J., Jeong H.Y., Ishihara T., Shin J., Kim G. (2016). A robust symmetrical electrode with layered perovskite structure for direct hydrocarbon solid oxide fuel cells: PrBa0.8Ca0.2Mn2O5+δ. J. Mater. Chem. A.

[54] Choi S., Yoo S., Kim J., Park S., Jun A., Sengodan S., Kim J., Shin J., Jeong H.Y., Choi Y. (2013). Highly efficient and robust cathode materials for low-temperature solid oxide fuel cells: PrBa_0.5_Sr_0.5_Co_2−x_Fe_x_O_5+δ_. Sci. Rep..

[55] Sengodan S., Choi S., Jun A., Shin T.H., Ju Y.W., Jeong H.Y., Shin J., Irvine J.T., Kim G. (2015). Layered oxygen-deficient double perovskite as an efficient and stable anode for direct hydrocarbon solid oxide fuel cells. Nat. Mater..

[56] Lv H., Lin L., Zhang X., Song Y., Matsumoto H., Zeng C., Ta N., Liu W., Gao D., Wang G. (2020). In Situ Investigation of Reversible Exsolution/Dissolution of CoFe Alloy Nanoparticles in a Co-Doped Sr_2_Fe_1.5_Mo_0.5_O_6-δ_ Cathode for CO_2_ Electrolysis. Adv. Mater..

[57] Adijanto L., Balaji Padmanabhan V., Holmes K.J., Gorte R.J., Vohs J.M. (2012). Physical and electrochemical properties of alkaline earth doped, rare earth vanadates. J. Solid State Chem..

[58] Petit C.T.G., Lan R., Cowin P.I., Irvine J.T.S., Tao S. (2011). Novel redox reversible oxide, Sr-doped cerium orthovanadate to metavanadate. J. Mater. Chem..

[59] Boulfrad S., Cassidy M., Irvine J.T.S. (2011). NbTi0.5Ni0.5O4 as anode compound material for SOFCs. Solid State Ion..

[60] Zenou V.Y., Fowler D.E., Gautier R., Barnett S.A., Poeppelmeier K.R., Marks L.D. (2016). Redox and phase behavior of Pd-substituted (La,Sr)CrO_3_ perovskite solid oxide fuel cell anodes. Solid State Ion..

[61] Lindenthal L., Rameshan R., Summerer H., Ruh T., Popovic J., Nenning A., Löffler S., Opitz A.K., Blaha P., Rameshan C. (2020). Modifying the Surface Structure of Perovskite-Based Catalysts by Nanoparticle Exsolution. Catalysts.

[62] Zhang S., Katz M.B., Dai S., Zhang K., Du X., Graham G.W., Pan X. (2017). New Atomic-Scale Insight into Self-Regeneration of Pt-CaTiO3 Catalysts: Incipient Redox-Induced Structures Revealed by a Small-Angle Tilting STEM Technique. J. Phys. Chem. C.

[63] Dai S., Zhang S., Katz M.B., Graham G.W., Pan X. (2017). In Situ Observation of Rh-CaTiO3 Catalysts during Reduction and Oxidation Treatments by Transmission Electron Microscopy. ACS Catal..

[64] Han H., Park J., Nam S.Y., Kim K.J., Choi G.M., Parkin S.S.P., Jang H.M., Irvine J.T.S. (2019). Lattice strain-enhanced exsolution of nanoparticles in thin films. Nat. Commun..

[65] Kim K.J., Han H., Defferriere T., Yoon D., Na S., Kim S.J., Dayaghi A.M., Son J., Oh T.S., Jang H.M. (2019). Facet-Dependent in Situ Growth of Nanoparticles in Epitaxial Thin Films: The Role of Interfacial Energy. J. Am. Chem. Soc..

[66] Neagu D., Kyriakou V., Roiban I.-L., Aouine M., Tang C., Caravaca A., Kousi K., Schreur-Piet I., Metcalfe I.S., Vernoux P. (2019). In Situ Observation of Nanoparticle Exsolution from Perovskite Oxides; from Atomic Scale Mechanistic Insight to Nanostructure Tailoring. ACS Nano.

[67] Zhu T., Troiani H., Mogni L.V., Santaya M., Han M., Barnett S.A. (2019). Exsolution and electrochemistry in perovskite solid oxide fuel cell anodes: Role of stoichiometry in Sr(Ti, Fe, Ni)O_3_. J. Power Sources.

[68] Zhu T., Troiani H.E., Mogni L.V., Han M., Barnett S.A. (2018). Ni-Substituted Sr(Ti,Fe)O_3_ SOFC Anodes: Achieving High Performance via Metal Alloy Nanoparticle Exsolution. Joule.

[69] Götsch T., Köpfle N., Grünbacher M., Bernardi J., Carbonio E.A., Hävecker M., Knop-Gericke A., Bekheet M.F., Schlicker L., Doran A. (2019). Crystallographic and electronic evolution of lanthanum strontium ferrite (La_0.6_ Sr_0.4_ FeO_3−δ_) thin film and bulk model systems during iron exsolution. Phys. Chem. Chem. Phys..

[70] Joo S., Kwon O., Kim K., Kim S., Kim H., Shin J., Jeong H.Y., Sengodan S., Han J.W., Kim G. (2019). Cation-swapped homogeneous nanoparticles in perovskite oxides for high power density. Nat. Commun..

[71] Joo S., Kwon O., Kim S., Jeong H.Y., Kim G. (2020). Ni-Fe Bimetallic Nanocatalysts Produced by Topotactic Exsolution in Fe deposited PrBaMn_1.7_Ni_0.3_O_5+δ_ for Dry Reforming of Methane. J. Electrochem. Soc..

[72] Joo S., Seong A., Kwon O., Kim K., Lee J.H., Gorte R.J., Vohs J.M., Han J.W., Kim G. (2020). Highly active dry methane reforming catalysts with boosted in situ grown Ni-Fe nanoparticles on perovskite via atomic layer deposition. Sci. Adv..

[73] Mao X., Foucher A.C., Stach E.A., Gorte R.J. (2020). Changes in Ni-NiO equilibrium due to LaFeO_3_ and the effect on dry reforming of CH_4_. J. Catal..

[74] Hamada I., Uozumi A., Morikawa Y., Yanase A., Katayama-Yoshida H. (2011). A density functional theory study of self-regenerating catalysts LaFe_(1−x)_M_(x)_O_(3−y)_ (M = Pd, Rh, Pt). J. Am. Chem. Soc..

[75] Raman A.S., Vojvodic A. (2020). Modeling Exsolution of Pt from ATiO3 Perovskites (A = Ca/Sr/Ba) Using First-Principles Methods. Chem. Mater..

[76] Mao X., Lin C., Graham G.W., Gorte R.J. (2020). A Perspective on Thin-Film Perovskites as Supports for Metal Catalysts. ACS Catal..

[77] Mao X., Foucher A.C., Montini T., Stach E.A., Fornasiero P., Gorte R.J. (2020). Epitaxial and Strong Support Interactions between Pt and LaFeO3 Films Stabilize Pt Dispersion. J. Am. Chem. Soc..

[78] Lin C., Foucher A.C., Ji Y., Curran C.D., Stach E.A., McIntosh S., Gorte R.J. (2019). “Intelligent” Pt Catalysts Studied on High-Surface-Area CaTiO3 Films. ACS Catal..

[79] Lin C., Foucher A.C., Ji Y., Stach E.A., Gorte R.J. (2020). Investigation of Rh–titanate (ATiO_3_) interactions on high-surface-area perovskite thin films prepared by atomic layer deposition. J. Mater. Chem. A.

[80] Helveg S., Sehested J., Rostrup-Nielsen J. (2011). Whisker carbon in perspective. Catal. Today.

[81] Papargyriou D., Miller D.N., Irvine J.T.S. (2019). Exsolution of Fe–Ni alloy nanoparticles from (La, Sr)(Cr, Fe, Ni) O 3 perovskites as potential oxygen transport membrane catalysts for methane reforming. J. Mater. Chem. A.

[82] McIntosh S., Gorte R.J. (2004). Direct hydrocarbon solid oxide fuel cells. Chem. Rev..

[83] Lin C., Jang J.B., Zhang L., Stach E.A., Gorte R.J. (2018). Improved Coking Resistance of “Intelligent” Ni Catalysts Prepared by Atomic Layer Deposition. ACS Catal..

[84] Mao X., Foucher A.C., Stach E.A., Gorte R.J. (2019). “Intelligent” Pt Catalysts Based on Thin LaCoO3 Films Prepared by Atomic Layer Deposition. Inorganics.

[85] Kishimoto H., Horita T., Yamaji K., Brito M.E., Xiong Y.-P., Yokokawa H. (2010). Sulfur poisoning on SOFC Ni anodes: Thermodynamic analyses within local equilibrium anode reaction model. J. Electrochem. Soc..

[86] Rostrup-Nielsen J., Hansen J., Helveg S., Christiansen N., Jannasch A.-K. (2006). Sites for catalysis and electrochemistry in solid oxide fuel cell (SOFC) anode. Appl. Phys. A.

[87] Brightman E., Ivey D., Brett D., Brandon N. (2011). The effect of current density on H2S-poisoning of nickel-based solid oxide fuel cell anodes. J. Power Sources.

[88] Song Y., Wang W., Ge L., Xu X., Zhang Z., Juliao P.S.B., Zhou W., Shao Z. (2017). Rational Design of a Water-Storable Hierarchical Architecture Decorated with Amorphous Barium Oxide and Nickel Nanoparticles as a Solid Oxide Fuel Cell Anode with Excellent Sulfur Tolerance. Adv. Sci..

[89] Sun Y., Li J., Zeng Y., Amirkhiz B.S., Wang M., Behnamian Y., Luo J. (2015). A-site deficient perovskite: The parent for in situ exsolution of highly active, regenerable nano-particles as SOFC anodes. J. Mater. Chem. A.

[90] Cui S.-H., Li J.-H., Zhou X.-W., Wang G.-Y., Luo J.-L., Chuang K.T., Bai Y., Qiao L.-J. (2013). Cobalt doped LaSrTiO3−δ as an anode catalyst: Effect of Co nanoparticle precipitation on SOFCs operating on H2S-containing hydrogen. J. Mater. Chem. A.

[91] Zhao C., Li Y., Zhang W., Zheng Y., Lou X., Yu B., Chen J., Chen Y., Liu M., Wang J. (2020). Heterointerface engineering for enhancing the electrochemical performance of solid oxide cells. Energy Environ. Sci..

[92] Liu Y., Jia L., Li J., Chi B., Pu J., Li J. (2020). High-performance Ni in-situ exsolved Ba(Ce_0.9_Y_0.1_)_0.8_Ni_0.2_O_3−δ_/Gd_0.1_Ce_0.9_O_1.95_ composite anode for SOFC with long-term stability in methane fuel. Compos. Part B Eng..

[93] Yang G., Su C., Chen Y., Tadé M.O., Shao Z. (2014). Nano La_0.6_Ca_0.4_Fe_0.8_Ni_0.2_O_3−δ_ decorated porous doped ceria as a novel cobalt-free electrode for “symmetrical” solid oxide fuel cells. J. Mater. Chem. A.

[94] Sun Y.-F., Zhou X.-W., Zeng Y., Amirkhiz B.S., Wang M.-N., Zhang L.-Z., Hua B., Li J., Li J.-H., Luo J.-L. (2015). An ingenious Ni/Ce co-doped titanate based perovskite as a coking-tolerant anode material for direct hydrocarbon solid oxide fuel cells. J. Mater. Chem. A.

[95] Lu J., Zhu C., Pan C., Lin W., Lemmon J.P., Chen F., Li C., Xie K. (2018). Highly efficient electrochemical reforming of CH_4_/CO_2_ in a solid oxide electrolyser. Sci. Adv..

[96] Madsen B.D., Kobsiriphat W., Wang Y., Marks L.D., Barnett S. (2007). SOFC anode performance enhancement through precipitation of nanoscale catalysts. ECS Trans..

[97] Thommy L., Joubert O., Hamon J., Caldes M.-T. (2016). Impregnation versus exsolution: Using metal catalysts to improve electrocatalytic properties of LSCM-based anodes operating at 600 °C. Int. J. Hydrog. Energy.

[98] Kobsiriphat W., Madsen B., Wang Y., Shah M., Marks L., Barnett S.A. (2010). Nickel-and ruthenium-doped lanthanum chromite anodes: Effects of nanoscale metal precipitation on solid oxide fuel cell performance. J. Electrochem. Soc..

[99] Vert V.B., Melo F.V., Navarrete L., Serra J.M. (2012). Redox stability and electrochemical study of nickel doped chromites as anodes for H2/CH4-fueled solid oxide fuel cells. Appl. Catal. B Environ..

[100] Sun Y.F., Li J.H., Cui L., Hua B., Cui S.H., Li J., Luo J.L. (2015). A-site-deficiency facilitated in situ growth of bimetallic Ni-Fe nano-alloys: A novel coking-tolerant fuel cell anode catalyst. Nanoscale.

[101] Wu N., Wang W., Zhong Y., Yang G., Qu J., Shao Z. (2017). Nickel-Iron Alloy Nanoparticle-Decorated K2NiF4 -Type Oxide as an Efficient and Sulfur-Tolerant Anode for Solid Oxide Fuel Cells. ChemElectroChem.

[102] Wang Z., Yin Y.-M., Yu Y., Song Y., Ma Z.-F., Yin J. (2018). Roles of Fe Ni nanoparticles and SrLaFeO4 substrate in the performance and reliability of a composite anode prepared through in-situ exsolution for intermediate temperature solid oxide fuel cells (I). Int. J. Hydrog. Energy.

[103] Park B.H., Choi G.M. (2014). Ex-solution of Ni nanoparticles in a La0.2Sr0.8Ti1−xNixO3−δ alternative anode for solid oxide fuel cell. Solid State Ion..

[104] Park B.H., Choi G.M. (2015). Effect of anode firing on the performance of lanthanum and nickel co-doped SrTiO3 (La_0.2_Sr_0.8_Ti_0.9_Ni_0.1_O_3−δ_) anode of solid oxide fuel cell. J. Power Sources.

[105] Arrivé C., Delahaye T., Joubert O., Gauthier G. (2013). Exsolution of nickel nanoparticles at the surface of a conducting titanate as potential hydrogen electrode material for solid oxide electrochemical cells. J. Power Sources.

[106] Ye L., Zhang M., Huang P., Guo G., Hong M., Li C., Irvine J.T., Xie K. (2017). Enhancing CO2 electrolysis through synergistic control of non-stoichiometry and doping to tune cathode surface structures. Nat. Commun..

[107] Bahout M., Managutti P.B., Dorcet V., Le Gal La Salle A., Paofai S., Hansen T.C. (2020). In situ exsolution of Ni particles on the PrBaMn2O5 SOFC electrode material monitored by high temperature neutron powder diffraction under hydrogen. J. Mater. Chem. A.

[108] Kwon O., Kim K., Joo S., Jeong H.Y., Shin J., Han J.W., Sengodan S., Kim G. (2018). Self-assembled alloy nanoparticles in a layered double perovskite as a fuel oxidation catalyst for solid oxide fuel cells. J. Mater. Chem. A.

[109] Wang Y., Liu T., Li M., Xia C., Zhou B., Chen F. (2016). Exsolved Fe–Ni nano-particles from Sr2Fe1.3Ni0.2Mo0.5O6 perovskite oxide as a cathode for solid oxide steam electrolysis cells. J. Mater. Chem. A.

[110] Xiao G., Wang S., Lin Y., Zhang Y., An K., Chen F. (2014). Releasing metal catalysts via phase transition: (NiO)_0.05_-(SrTi_0.8_Nb_0.2_O_3_)_0.95_ as a redox stable anode material for solid oxide fuel cells. ACS Appl. Mater. Interfaces.

[111] Kyriakou V., Neagu D., Zafeiropoulos G., Sharma R.K., Tang C., Kousi K., Metcalfe I.S., van de Sanden M.C.M., Tsampas M.N. (2019). Symmetrical Exsolution of Rh Nanoparticles in Solid Oxide Cells for Efficient Syngas Production from Greenhouse Gases. ACS Catal..

[112] Monteiro N.K., Noronha F.B., da Costa L.O.O., Linardi M., Fonseca F.C. (2012). A direct ethanol anode for solid oxide fuel cell based on a chromite-manganite with catalytic ruthenium nanoparticles. Int. J. Hydrogen Energy.

[113] Liao Y., Bierschenk D.M., Barnett S.A., Marks L.D. (2011). Operational Inhomogeneities in La0.9Sr0.1Ga0.8Mg0.2O3-δ Electrolytes and La_0.8_Sr_0.2_Cr_0.82_Ru_0.18_O_3−δ_-Ce_0.9_Gd_0.1_O_2−δ_ Composite Anodes for Solid Oxide Fuel Cells. Fuel Cells.

[114] Kobsiriphat W., Madsen B.D., Wang Y., Marks L.D., Barnett S.A. (2009). La_0.8_Sr_0.2_Cr_1−x_Ru_x_O_3−δ_–Gd_0.1_Ce_0.9_O_1.95_ solid oxide fuel cell anodes: Ru precipitation and electrochemical performance. Solid State Ion..

[115] Bierschenk D.M., Potter-Nelson E., Hoel C., Liao Y., Marks L., Poeppelmeier K.R., Barnett S.A. (2011). Pd-substituted (La,Sr)CrO_3_−δ–Ce_0.9_Gd_0.1_O_2−δ_ solid oxide fuel cell anodes exhibiting regenerative behavior. J. Power Sources.

[116] Marcucci A., Zurlo F., Sora I.N., Placidi E., Casciardi S., Licoccia S., Di Bartolomeo E. (2019). A redox stable Pd-doped perovskite for SOFC applications. J. Mater. Chem. A.

[117] Shin T.H., Okamoto Y., Ida S., Ishihara T. (2012). Self-recovery of Pd nanoparticles that were dispersed over La(Sr)Fe(Mn)O_3_ for intelligent oxide anodes of solid-oxide fuel cells. Chem. Eur. J..

[118] Glaser R., Zhu T., Troiani H., Caneiro A., Mogni L., Barnett S. (2018). The enhanced electrochemical response of Sr(Ti_0.3_Fe_0.7_Ru_0.07_)O_3−δ_ anodes due to exsolved Ru–Fe nanoparticles. J. Mater. Chem. A.

[119] Chanthanumataporn M., Hui J., Yue X., Kakinuma K., Irvine J.T.S., Hanamura K. (2019). Electrical reduction of perovskite electrodes for accelerating exsolution of nanoparticles. Electrochim. Acta.

[120] Sun Y.-F., Li J.-H., Wang M.-N., Hua B., Li J., Luo J.-L. (2015). A-site deficient chromite perovskite with in situ exsolution of nano-Fe: A promising bi-functional catalyst bridging the growth of CNTs and SOFCs. J. Mater. Chem. A.

[121] Qi H., Yang T., Li W., Ma L., Hu S., Shi W., Sabolsky E.M., Zondlo J.W., Hart R., Hackett G.A. (2019). Reversible In-Situ Exsolution of Fe Catalyst in La_0.5_Sr_1.5_Fe_1.5_Mo_0.5_O_6−δ_ Anode for SOFCs. ECS Trans..

[122] Qi H., Xia F., Yang T., Li W., Li W., Ma L., Collins G., Shi W., Tian H., Hu S. (2020). In Situ Exsolved Nanoparticles on La_0.5_Sr_1.5_Fe_1.5_Mo_0.5_O_6−δ_ Anode Enhance the Hydrogen Oxidation Reaction in SOFCs. J. Electrochem. Soc..

[123] Wang W., Zhu C., Xie K., Gan L. (2018). High performance, coking-resistant and sulfur-tolerant anode for solid oxide fuel cell. J. Power Sources.

[124] Li J., Wei B., Cao Z., Yue X., Zhang Y., Lu Z. (2018). Niobium Doped Lanthanum Strontium Ferrite as A Redox-Stable and Sulfur-Tolerant Anode for Solid Oxide Fuel Cells. ChemSusChem.

[125] Chen X., Ni W., Wang J., Zhong Q., Han M., Zhu T. (2018). Exploration of Co-Fe alloy precipitation and electrochemical behavior hysteresis using Lanthanum and Cobalt co-substituted SrFeO_3−δ_ SOFC anode. Electrochim. Acta.

[126] Lai K.-Y., Manthiram A. (2018). Self-Regenerating Co–Fe Nanoparticles on Perovskite Oxides as a Hydrocarbon Fuel Oxidation Catalyst in Solid Oxide Fuel Cells. Chem. Mater..

[127] Yang C., Li J., Lin Y., Liu J., Chen F., Liu M. (2015). In situ fabrication of CoFe alloy nanoparticles structured (Pr_0.4_Sr_0.6_)_3_(Fe_0.85_Nb_0.15_)_2_O_7_ ceramic anode for direct hydrocarbon solid oxide fuel cells. Nano Energy.

[128] Yang C., Yang Z., Jin C., Xiao G., Chen F., Han M. (2012). Sulfur-tolerant redox-reversible anode material for direct hydrocarbon solid oxide fuel cells. Adv. Mater..

[129] Yang Y., Wang Y., Yang Z., Lei Z., Jin C., Liu Y., Wang Y., Peng S. (2019). Co-substituted Sr2Fe1.5Mo0.5O6-δ as anode materials for solid oxide fuel cells: Achieving high performance via nanoparticle exsolution. J. Power Sources.

[130] Xi X., Cao Z.-S., Shen X.-Q., Lu Y., Li J., Luo J.-L., Fu X.-Z. (2020). In situ embedding of CoFe nanocatalysts into Sr_3_FeMoO_7_ matrix as high-performance anode materials for solid oxide fuel cells. J. Power Sources.

